# Effects of *Streptococcus sanguinis* Bacteriocin on Cell Surface Hydrophobicity, Membrane Permeability, and Ultrastructure of *Candida* Thallus

**DOI:** 10.1155/2015/514152

**Published:** 2015-04-29

**Authors:** Shengli Ma, Yingnan Zhao, Xue Xia, Xue Dong, Wenyu Ge, Hui Li

**Affiliations:** ^1^Department of Stomatology, Hospital of Heilongjiang Province, Harbin 150036, China; ^2^University of Jiamusi, Jiamusi 154002, China; ^3^The 2nd Affiliated Hospital of Harbin Medical University, Harbin 150001, China

## Abstract

*Candida albicans* (C.a) and *Candida tropicalis* (C.t) were treated with *Streptococcus sanguinis* bacteriocin (S.s bacteriocin), respectively; the bacteriostatic dynamics of S.s bacteriocin, their effects on cell surface hydrophobicity, leakage of inorganic phosphorus and macromolecular substance, cytosolic calcium concentration, and ultrastructure changes of *Candida* thallus were detected and analyzed. The results showed that inhibitory effect of S.s bacteriocin on C.a and C.t reached peak level at 24 h, the cell-surface hydrophobicity decreased significantly (*P* < 0.05) after S.s bacteriocin treatment, and there was leakage of cytoplasmic inorganic phosphorus and macromolecular substance from C.a and C.t; cytosolic calcium concentration decreased greatly. After 24 h treatment by S.s bacteriocin, depressive deformity and defect could be found in the cell surface of C.a and C.t; the thallus displayed irregular forms: C.a was shrunken, there was unclear margins abutting upon cell wall and cell membrane, nucleus disappeared, and cytoplasm was inhomogeneous; likewise, C.t was first plasmolysis, and then the cytoplasm was shrunk, the ultrastructure of cell wall and cell membrane was continuously damaged, and the nucleus was karyolysis. It was illustrated that S.s bacteriocin had similar antifungal effect on C.a and C.t; their cell surface hydrophobicity, membrane permeability, and ultrastructure were changed significantly on exposure to S.s bacteriocin.

## 1. Introduction

With the wide application of broad-spectrum antibiotics, glucocorticoid, and immunosuppressive agents and the increasing of tumor cases by chemotherapy or radiotherapy, organ transplantation patients, and HIV/AIDS patients, there is an upward tendency of morbidity and mortality of deep fungal infection [1, 2].* Candida* is the main pathogen causing deep fungal infection in clinic; the highest rate of infection occurred at 5 fungi strains:* Candida albicans* (C.a),* Candida glabrata* (C.g),* Candida tropicalis* (C.t),* Candida parapsilosis* (C.p), and* Candida krusei* (C.k) [3]. The narrow antimicrobial spectrum, side effects, and drug resistances of the existing anti-*Candida* drugs prompt an urgent research for new inhibitors of* Candida* [4]. It was reported that plenty of virulence factors constituted the main sources of pathogenicity in* Candida*, including cell surface hydrophobicity, hemolytic activity, and adhesiveness; among them, hydrophobic interaction, as one nonspecificity adherence mechanism, played an important role in initial bacterial adherence to teeth and oral epithelial cells [5]; also it has been demonstrated to take part in the bacteria aggregation process [6]. The effective control of* Candida* is an urgent problem to be solved. In the recent years, bacteriocin-like substances had been extracted from culture medium of* Streptococcus sanguinis*; they were called* Streptococcus sanguinis* bacteriocin (S.s bacteriocin) [7–9]. It had been found that the S.s bacteriocin could suppress the growth of C.a and C.t effectively and resulted in their morphological changes [8]. So far, the antifungal mechanisms of S.s bacteriocin are still unknown. In this study, the relationships among S.s bacteriocin antifungal activity and time variation, effects of S.s bacteriocin on cell surface hydrophobicity, membrane permeability, and ultrastructure of* Candida* thallus were analyzed, and the bacteriostatic mechanisms were explored.

## 2. Materials and Methods

### 2.1. Strains and Mediums

Standard strain ATCC10556 of S.s was purchased from state key laboratory of oral diseases in West China College of Stomatology Sichuan University, standard strain ATCC10231 of C.a and ATCC13803 of C.t were purchased from Shanghai Fu Xiang Biotechnology Co. Ltd., Brain Heart Infusion Broth (BHI) and Yeast Extract Peptone Dextrose Medium (YPD) were purchased from OXOID England, and Columbia Blood Agar Base Medium and* Candida* Chromogenic Medium were purchased from CHROMagar France.

### 2.2. Experimental Methods

#### 2.2.1. Extraction of S.s Bacteriocin

Standard strain of S.s was identified and pure cultured; then they were inoculated to 4 L BHI culture medium and anaerobic cultured at 37°C for 72 h. Medium of S.s was ultracentrifuged at low temperature (10000 r/min, 4°C) for 30 min after anaerobic culture; bacteria precipitation was collected, washed 3 times, and divided into 10 parts, resuspended in 5 mL PBS, respectively; after repeated freezing and thawing, cell crushing, and centrifuging, the supernatants were salted out by ammonium sulphate gradients method and desalted by sephadex G-25; the collections were dialysed, condensed, lyophilized, and cryopreserved at −80°C.

#### 2.2.2. Fungistatic Dynamics of S.s Bacteriocin on C.a and C.t

Characteristics of fungistatic dynamics of S.s bacteriocin were observed by liquid antifungal culture [10]; test* Candida* of C.a and C.t in logarithmic growth phase were prepared with a concentration of 10^6^ cfu/mL (*A* = 0.001~0.002) by YPD culture medium; then they were cocultured with 1 g/L S.s bacteriocin in 96-well plate at 37°C, 150 r/min. Absorbance (*A*) of samples was detected every 3 h by automated ELIASA (BioTek, USA) at 630 nm wave length. The group without S.s bacteriocin was used as control. Fungistatic vitality was calculated using the method of Hultmark [11]: *U* = $\sqrt{(A_{0}-A)/A_{0}}$ (*A*_0_ referred to the absorbance in control group).

#### 2.2.3. Effects of S.s Bacteriocin on Cell Surface Hydrophobicity of C.a and C.t

Microbial adhesion to hydrocarbons (MATH) [12] and ultraviolet-spectrophotometer assay were used to measure the cell surface hydrophobicity. Media of C.a and C.t in logarithmic growth phase were centrifuged at 3000 r/min for 20 min; the precipitation was collected, washed 3 times with PBS, and adjusted to OD 0.5 by ultraviolet-spectrophotometer at 600 nm wave length; the suspensions were then divided into 3 groups with a volume of 3 mL suspensions in each group; 500 *μ*L of PBS, 1 g/L S.s bacteriocin, and 5.25% chlorhexidine were added to each group, respectively. The 3 groups were cultured at 37°C for 24 h; then they were mixed with 400 *μ*L hexadecane solutions and vortexed for 60 s; the mixture was left to stand and demixed to oil and water layers; oil layers were discarded and absorbance of water layers at 600 nm wave length was measured to determine the value of optical density (OD); the value of cell surface hydrophobicity (CSH) was calculated according the formula CSH = (blank OD_600 nm_ − treatment OD_600 nm_)/blank OD_600 nm_ × 100%. The test in each group was repeated 15 times.

#### 2.2.4. Effects of S.s Bacteriocin on Phosphorus Leakage of C.a and C.t

Media of C.a and C.t in logarithmic growth phase were centrifuged and the precipitation was collected; they were resuspended in 1.0 mmol/L Tris-Hcl buffer (pH 7.0), respectively. In the treatment group, the suspensions, Tris-Hcl buffer, and 1 g/L S.s bacteriocin were mixed; the final concentration of* Candida* was 10^6^ cfu/mL. In the control group, S.s bacteriocin was replaced with sterile distilled water. Each group was cultured at 37°C; 2 mL culture medium was collected every 3 h from each group; then they were centrifuged. Phosphorus content in the supernatant was examined by ammonium molybdate colorimetry, specific as follows: 2 mL solutions were collected from treatment and control group and then they were added to 20 mL stoppered test tube, respectively, mixed with 2 mL ammonium molybdate solutions, and stood for a few seconds; then they were mixed with 1 mL 0.5% hydroquinone and 1 mL 20% sodium sulfite. The mixture was standing for 30 min; absorbance of mixtures at 660 nm wave length was measured to determine optical density (OD) value.

#### 2.2.5. Effects of S.s Bacteriocin on UV-Absorbing Substance Permeability of C.a and C.t

Test* Candida* of C.a and C.t in logarithmic growth phase were prepared with a concentration of 10^6^ cfu/mL by 0.1 mol/L phosphate buffer (pH 7.4); then they were cocultured with 1 g/L S.s bacteriocin at 37°C for 3, 6, 9, 12, 15, 18, and 24 h, respectively. In another group, S.s bacteriocin was not added; test* Candida* was ruptured by adding 2 mg/mL TritonX-100 at each sampling time. The group without S.s bacteriocin treatment was used as control. Absorbance (*A*) of samples at 260 nm wave length was detected. UV-absorbing substance permeability was calculated according the formula PDL = *A*/*A*_*t*_ × 100 (*A* referred to the absorbance with or without S.s bacteriocin treatment; *A*_*t*_ referred to the absorbance of TritonX-100 treatment).

#### 2.2.6. Effects of S.s Bacteriocin on Cytosolic Calcium Concentration of C.a and C.t

Test* Candida* of C.a and C.t in logarithmic growth phase were prepared with a concentration of 10^6^ cfu/mL (*A* = 0.001~0.002) by YPD culture medium; 300 *μ*L suspensions of test* Candida* were placed on cover glass and then they were cultured in sterilized 6-well plate at 37°C for 4 h, so the* Candida* was attached to the cover glass; then they were cocultured with 300 *μ*L 1 g/L S.s bacteriocin for another 24 h. In the control group, S.s bacteriocin was replaced with deionized water. In the end of culture, the medium was discarded and washed with 1 mL PBS buffer 2 times, each time for 10 s; then 1 *μ*L preheated Fluo-3 AM was added and cultured at 37°C for 45 min; finally, the medium was discarded, washed with 1 mL PBS buffer 3 times, and cultured at 37°C for 15 min to make sure all the Fluo-3 AM were converted to Fluo-3 and then sealed with 50 *μ*L antifluorescent quencher.

#### 2.2.7. Ultrastructure of C.a and C.t Observed by Scanning Electron Microscope

Test* Candida* of C.a and C.t in logarithmic growth phase were prepared with a concentration of 10^6^ cfu/mL (*A* = 0.001~0.002) by YPD culture medium; then they were treated with 1 g/L S.s bacteriocin, respectively; in the control group, S.s bacteriocin was replaced with sterile distilled water. Each group was cultured at 37°C for 24 h; in the end of culture, medium was centrifuged (3000 r/min) for 5 min; the precipitation was collected, washed and fixed for specimens preparation, after gradient dehydration, air dry and gold spray, the specimens were observed under a SEM to evaluate the morphology changes caused by S.s bacteriocin.

#### 2.2.8. Ultrastructure of C.a and C.t Observed by Transmission Electron Microscope

Test* Candida* of C.a and C.t in logarithmic growth phase were prepared with a concentration of 10^6^ cfu/mL (*A* = 0.001~0.002) by YPD culture medium; then they were treated with 1 g/L S.s bacteriocin, respectively; in the control group, S.s bacteriocin was replaced with sterile distilled water. Each group was cultured at 37°C for 24 h; in the end of culture, medium was centrifuged (3000 r/min) for 5 min; the precipitation was collected, washed 2 times with sterile distilled water, fixed subsequently by 3% Glutaral for 12 h and 1% osmic acid for 1.5 h, rinsed by sterile distilled water 2 times, gradient-dehydrated by acetone, saturated by Epon 812^#^ at 35°C for 3 h, and embedded at 60°C for 36 h; after ultrathin section, lead and uranium staining, the specimens were observed under a TEM to evaluate the morphology changes caused by S.s bacteriocin.

### 2.3. Statistics and Analysis

*X*-test, one-way analysis of variance, and logistic analysis were conducted using SPSS 16.0. *P* = 0.05 was considered as test standard; *P* < 0.05 indicated that the result had the statistical significance.

## 3. Results

### 3.1. Fungistatic Dynamics of S.s Bacteriocin on C.a and C.t

**Figure 1:**
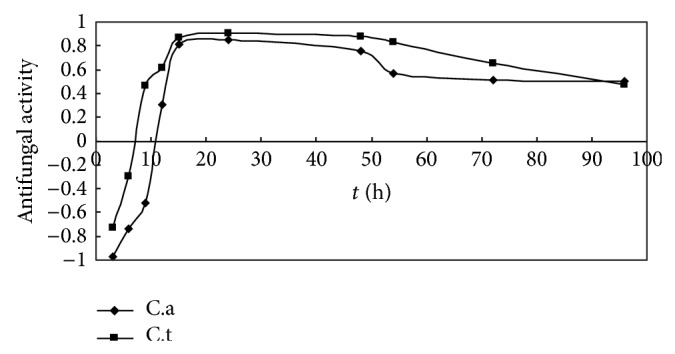
Antifungal dynamics of* Streptococcus sanguinis* bacteriocin on C.a and C.t.

Effects of S.s bacteriocin on* Candida* activity in relation to the treatment time were shown in Figure 1; the antifungal activity of S.s bacteriocin increased logarithmically at 0–15 h and reached the climax at 24 h and then began to decrease. Proximity of antifungal dynamics of S.s bacteriocin for C.a and C.t was 0.910, indicating that characteristics of fungistatic dynamics of S.s bacteriocin were quite similar between C.t and C.a.

### 3.2. Effects of S.s Bacteriocin on Cell Surface Hydrophobicity of C.a and C.t

The optical density (OD) value increased significantly in test group compared with control group (*P* < 0.01), indicating that the hydrophobicity decreased significantly, while there was no significant difference between test and positive control group (*P* > 0.05) (Table 1).

### 3.3. Effects of S.s Bacteriocin on Phosphorus Leakage of C.a and C.t

**Figure 2:**
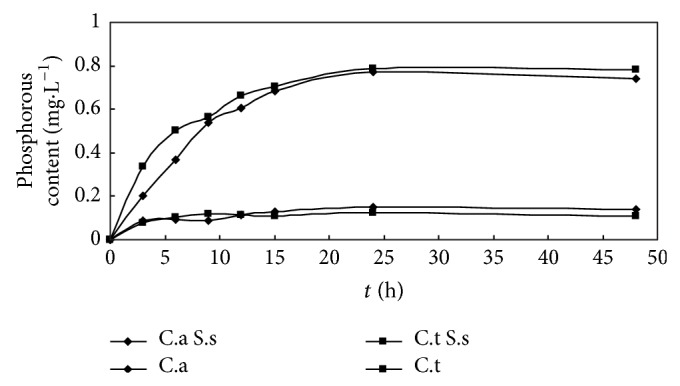
Effect of* Streptococcus sanguinis* bacteriocin on phosphorous leakage of C.a and C.t.

The quantity of phosphorus leakage of C.a and C.t after S.s bacteriocin treatment was significantly higher than the group without S.s bacteriocin treatment; the quantity changed greatly in 0–15 h, became slow in 15–24 h, and reached the top level at 24 h and then became stable. The quantity of phosphorus leakage of C.a and C.t was 0.775 mg/L and 0.785 mg/L, respectively. Without S.s bacteriocin treatment, the quantity of phosphorus leakage was little (Figure 2).

### 3.4. Effects of S.s Bacteriocin on UV-Absorbing Substance Permeability of C.a and C.t

**Figure 3:**
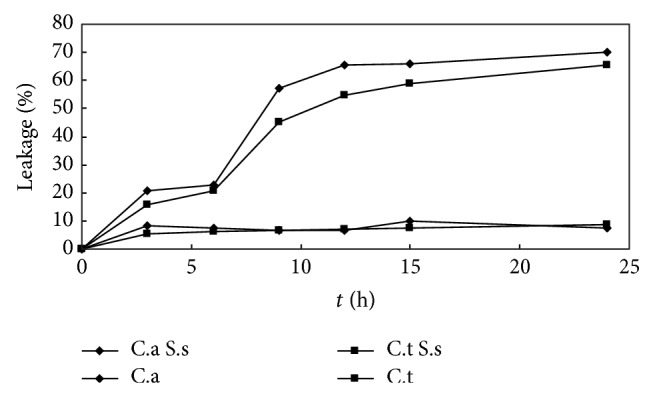
Permeability of UV-absorbing substance in C.a and C.t treated with and without* Streptococcus sanguinis* bacteriocin.

The UV-absorbing substance permeability of C.a and C.t was increasing slowly in the first 6 h after S.s bacteriocin treatment; with the time extending, the permeability increased greatly in 6–12 h; after 12 h, the trend returned to be slow, while, without S.s bacteriocin treatment, UV-absorbing substance permeability was little (Figure 3).

### 3.5. Effects of S.s Bacteriocin on Cytosolic Calcium Concentration of C.a and C.t

**Figure 4:**
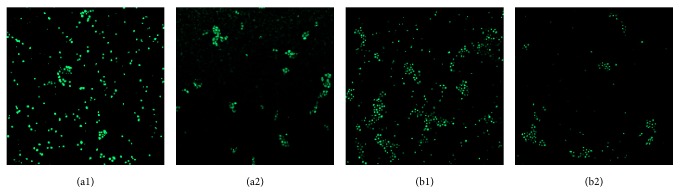
Ca^2+^ fluorescence intensity of C.a and C.t in the control group and the treatment group. (a1) C.a in control group, 24 h; (a2) C.a in treatment group, 24 h; (b1) C.t in control group, 24 h; (b2) C.t in treatment group, 24 h.

Fluorescence intensity of calcium ion was observed by laser confocal microscopy and analyzed by ImageJ software; the relative value of fluorescence intensity was calculated by the following formula: variation percentage of calcium ion [Ca^2+^i] = (*F*_0_ − *F*)/*F*_0_ × 100%; *F* represented fluorescence intensity after S.s bacteriocin treatment; *F*_0_ represented fluorescence intensity of control group. The result showed that cytosolic calcium concentration and Ca^2+^i were decreased significantly in the test group (Figure 4 and Table 2).

### 3.6. Ultrastructure of C.a and C.t Observed by Scanning Electron Microscope

**Figure 5:**
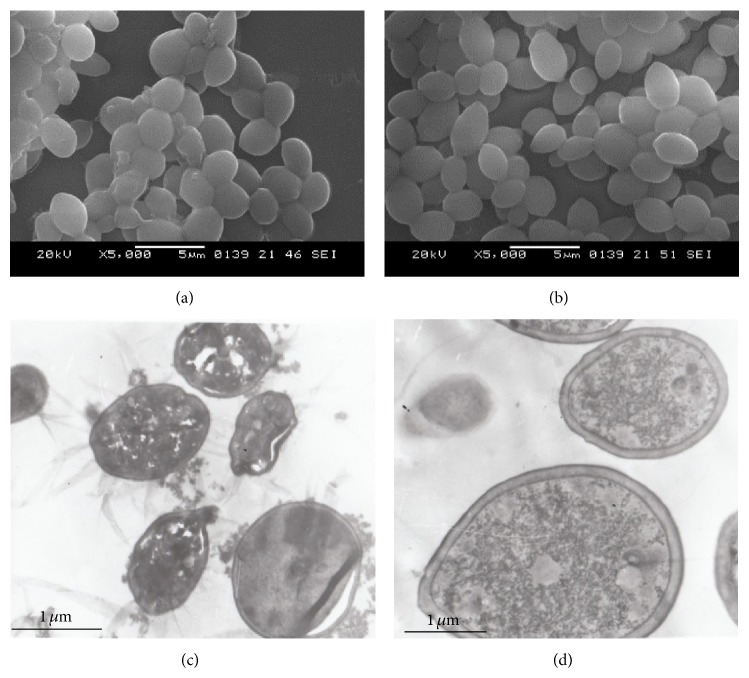
Effect of* Streptococcus sanguinis* bacteriocin on ultramicrostructure of C.a. (a) C.a after 24 h S.s bacteriocin treatment, SEM observation. It was identified that disc-like depressions of different depths were present in the surfaces; irregular thallus was identified. (b) C.a in the control group, SEM observation. They had a full shape and smooth surface. (c) C.a after 24 h S.s bacteriocin treatment, TEM observation. C.a thallus was deformed and shrunk; cell wall and membranes became thin and the boundary was unclear, plasmolysis and nucleus disappeared; cytoplasm was nonuniform. (d) C.a in the control group, TEM observation. They had a full shape and smooth surface; nuclear membrane was complete.

**Figure 6:**
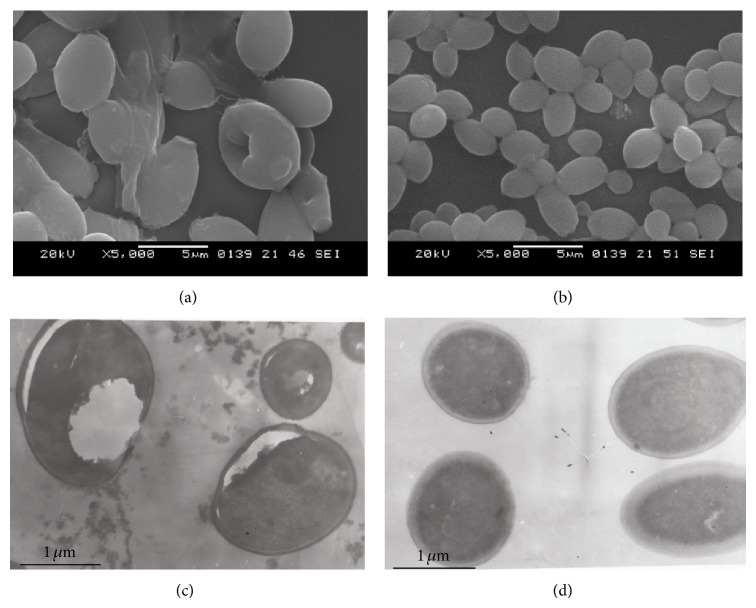
Effect of* Streptococcus sanguinis* bacteriocin on ultramicrostructure of C.t. (a) C.t after 24 h S.s bacteriocin treatment, SEM observation. There were not only depressions but also apophysis with different sizes in the surfaces. (b) C.t in the control group, SEM observation. They had a full shape and smooth surface. (c) C.t after 24 h S.s bacteriocin treatment, TEM observation. Cell wall of thallus became relaxed; there were plasmolysis, cytoplasm pycnosis, continuous destruction of cell wall and membrance, karyolysis, and cavitation of cytoplasm. (d) C.t in the control group, TEM observation. They had a full shape and smooth surface; nuclear membrane was complete.

It was identified that disc-like depressions of different depths were present in the surfaces of C.a after S.s bacteriocin treatment, and the diameter of the depressions was 0.1–0.5 *μ*m; 2–4 depressions were occasionally observed; irregular thallus was identified (Figure 5(a)). There were not only depressions but also apophysis with different sizes in the surfaces of C.t after S.s bacteriocin treatment; the diameter of the depressions was 0.5–1.6 *μ*m (Figure 6(a)). There were round or oval thalli in the control group cultures of C.a and C.t; they had a full shape and smooth surface (Figures 5(b) and 6(b)).

### 3.7. Ultrastructure of C.a and C.t Observed by Transmission Electron Microscope

The C.a thallus was deformed and shrunk after S.s bacteriocin treatment; cell wall and membranes became thin and the boundary was unclear; plasmolysis and nucleus disappeared; cytoplasm was nonuniform (Figure 5(c)); cell wall of C.t thallus became relaxed; there were plasmolysis, cytoplasm pycnosis, continuous destruction of cell wall and membrane, karyolysis, and cavitation of cytoplasm (Figure 6(c)), while, in control group, thalli were of round or oval shape, the diameter was 2–5 *μ*m, and they had a full shape and smooth surface; free ribosomes were abundant in the entocyte; nuclear membrane was complete; there was large fat body and glycogen granule in the cytoplasm (Figures 5(d) and 6(d)).

## 4. Discussions

Bacteriocins are generated by ribosome synthesis mechanism in metabolic processes of some bacteria; they are a kind of proteins, polypeptides, or precursor polypeptides [13].* Streptococcus sanguinis* (*S. sanguinis*) is the dominant bacteria in a healthy oral cavity and it is generally accepted that an antibacterial substance generated by* S. sanguinis* has an inhibitory effect on putative periodontal pathogens; in our previous studies, it was found that there were significant inhibitory effects of the intracellular proteins extracted from S. sanguinis on pathogenic bacteria (*P. intermedia* and* P. gingivalis*), fungi (*C. albicans* and* C. tropicalis*), and the biofilms formed by them; the growth curves and morphology of* C. albicans* and* C. tropicalis* were altered following treatment with the intracellular proteins, resulting in disc-like depressions in the surfaces of the fungal spores and mycelia [8]. In this study, S.s bacteriocin was extracted from culture medium of* Streptococcus sanguinis* by being ultracentrifuged at low temperature, ultrasonication, and so on. Fungistatic dynamics study showed that antifungal dynamics of S.s bacteriocin for C.a and C.t reached the climax at 24 h, and characteristics of fungistatic dynamics of S.s bacteriocin were quite similar between C.t and C.a.

The effects of S.s bacteriocin on cell surface hydrophobicity of C.a and C.t showed that S.s bacteriocin could result in the hydrophobicity decrease of C.a and C.t. It was reported that cell surface hydrophobicity played an important role in the initial adhesion of* Candida* to oral epithelium; the stronger the surface hydrophobicity the* Candida* had, the stronger the adhesion it had [14]. Cell surface hydrophobicity was related to the cell surface structure (protein, lipoteichoic acid, capsule, etc.); the change of hydrophobicity could reflect the biochemical modification of the cell wall.

There were some macromolecules with relative molecular mass of 190 kD in hydrophobic cell; they were called surface proteantigen, and they were found in the bacterial cell walls; without these proteins, the hydrophobicity was decreased [15]. It was reported that hydrophobic protein in the* Candida* surface could bind to hydrophobic groups; the formation ability of* Candida* biomembrane had a positive correlation with cell surface hydrophobicity [16]. In this study, S.s bacteriocin resulted in the decrease of cell surface hydrophobicity in C.a and C.t. It could be suspected that hydrophobic proteins in the cell surface were destroyed by S.s bacteriocin, and their combination to host ligands was blocked by S.s bacteriocin; adhesive attraction of C.a and C.t was inhibited by the decrease of cell surface hydrophobic interaction, so the interactive network between* Candida* and host histiocyte was cut off, the formation of* Candida* biomembrane was hampered, and the pathogenicity of* Candida* was decreased.

In the process of cellular metabolism, there were different materials that went into/out of the cell, including some ions, micromolecules, macromolecules, and particulate matters. In normal cases, the penetration to cell membrane of these materials could be divided to 3 ways: in the first way, water-solubility micromolecule could penetrate the cell membrane by the mediation of membrane protein, for example, inorganic phosphorus and amine acid; in the second way, macromolecule and some particulate matters proceeded the transmembrane transport through a particular way, for example, protein, Ca^2+^; in the third way, micromolecule with less polarity could penetrate the cell membrane by physical diffusion, for example, O_2_, hydrone [17]. In this study, the quantity of phosphorus leakage of C.a and C.t after S.s bacteriocin treatment changed greatly in 0–15 h and reached the top level at 24 h.

UV-absorbing substance permeability of C.a and C.t was increasing slowly in the first 6 h after S.s bacteriocin treatment; with the time extending, the permeability increased greatly, while, without S.s bacteriocin treatment, UV-absorbing substance permeability was little. Fluo-3 AM was hydrolyzed to free acid by intracellular hydrolase after entering cells; their conjugates with calcium ion could generate specific fluorescence under excitation light, and the intensity of fluorescence signals was changed following the concentration of calcium ion; the result showed that cytosolic calcium concentration and Ca^2+^i were decreased significantly in the test group.

It could be found that there were leakages of inorganic phosphorus, UV-absorbing substance, and decrease of intracellular calcium ion. In normal cases, the micromolecule and macromolecule could not penetrate the cell membrane, but when the cell membrane permeability was changed, there might be the leakages of cell contents.

The roles of S.s bacteriocin could be the following. (1) The cell membrane structures might be destroyed by S.s bacteriocin, resulting in the formation of channels in the cell membrane, so the cell membrane permeability was changed; the micromolecule and macromolecule could be released from thallus of* Candida*. (2) The main structure of cell membrane was constituted by phospholipid bilayer; S.s bacteriocin could bind to some specific compositions in the bilayer; integrity of cell membrane was broken, so the cell membrane permeability was changed; the transport of micromolecule and macromolecule in thallus of* Candida* became abnormal. (3) Ergosterol was an important component of fungal cell membrane; it could stabilize the structure of cell membrane and decrease their fluidity [18]; with the treatment of S.s bacteriocin, leakages of inorganic phosphorus, UV-absorbing substance in C.a and C.t was increased; it might hamper the synthesis of ergosterol; thus the stability of cell membrane was broken and the fluidity was increased. Finally, cell membrane ruptured and turned to death.

In the present, the main mechanism of antifungal agents was as follows: the destruction of fungal cell wall, interference on fungal cell membrane, inhibition of protein synthesis, and influence on mitochondria ATP synthetase [18]. In this study, the cell surface morphology and internal structure of C.a and C.t were changed after S.s bacteriocin treatment. It could be speculated that the cell membrane permeability of* Candida* was changed by the effect of S.s bacteriocin; many of them were accumulated in the thallus of* Candida* and combined specifically to molecules with cell wall synthesis function, so the cell wall synthesis of* Candida* was affected and resulted in deformation and collapse of cell wall, with various depressions appearing.

There were cytoplasm pycnosis, plasmolysis, nucleus destruction, disappearance, and vacuolization in C.a and C.t after 24 h treatment of S.s bacteriocin. It could be speculated that S.s bacteriocin could cause the organelle damage of* Candida* thallus and then affect the synthesis and expression of protein and DNA in* Candida*, finally resulting in transfer interruption of material, energy, and information, and the cell turned to death. Besides, S.s bacteriocin could attach to the surface of* Candida* cell wall by electrostatic attraction; the enzyme synthesis of cell wall was inhibited and the cell wall structure was destroyed, so depression, defect, and fracture could be found in the surface of cell wall.

In conclusion, S.s bacteriocin could not only cause the same fungistatic dynamics of C.a and C.t, but also result in the decrease of hydrophobicity in the cell surface, the cell membrane permeability, and ultrastructure changes of* Candida* thallus. The results could provide references for fungistatic mechanisms studies.

## Figures and Tables

**Table 1:** Hydrophobicity of *Streptococcus sanguinis* bacteriocin on single and mixed cultured C.a and C.t.

Group	Materials add-in (*μ*L)			*n*	Hydrophobicity (%)
	Deionized water	Sanguicin	Chlorhexidine		
C.a (10^6^ cfu/mL)	500	0	0	15	63.87 ± 2.92^∗^
	0	500	0	15	10.00 ± 2.62^∗^
	0	0	500	15	10.67 ± 1.95^∗^

C.t (10^6^ cfu/mL)	500	0	0	15	61.60 ± 4.91^∗^
	0	500	0	15	5.87 ± 245^∗^
	0	0	500	15	6.93 ± 2.37^∗^

*Note*. ^∗^Significant difference compared with control group (*P* < 0.05).

**Table 2:** Fluorescence intensity change and percentage of Ca^2+^ of *Streptococcus sanguinis* bacteriocin on single and mixed culture C.a and C.t (10^6^ cfu/mL).

Group	Materials add-in (*μ*L)		*F*_0_	*F*	[Ca^2+^i]
	Deionized water	Sanguicin			
C.a	300	0	43.30 ± 0.68	44.85 ± 0.57	3.59 ± 1.34
	0	300	43.20 ± 0.77	24.60 ± 0.67^∗^	43.30 ± 1.80^∗∗^

C.t	300	0	41.19 ± 1.01	41.87 ± 0.82	1.68 ± 2.69
	0	300	41.43 ± 0.82	21.67 ± 0.88^∗^	47.67 ± 2.78^∗∗^

*Note*. ^∗^Significant difference compared with control group (*P* < 0.05).

^∗∗^Significant difference compared with control group (*P* < 0.01).
